# Development of the Swedish anticholinergic burden scale (Swe-ABS)

**DOI:** 10.1186/s12877-023-04225-1

**Published:** 2023-08-25

**Authors:** Tanja Rube, Astrid Ecorcheville, Elisabet Londos, Sara Modig, Per Johansson

**Affiliations:** 1 Present Address: Memory Clinic, Ängelholm, SE-262 52 Sweden; 2 Cognitive Disorders Research Unit, Department of Clinical Sciences, Lund University, Malmö, Sweden; 3 Clinical Pharmacology, Lund, SE-221 85 Sweden; 4 Division of Clinical Geriatrics, Department of Neurobiology, Care Sciences and Society, Karolinska Institute, Stockholm, Sweden; 5 Department of Clinical Sciences, Lund University, Malmö, Sweden; 6 Primary Healthcare, Skåne County, Lund, Sweden; 7 Department of Medicines Management and Informatics in Skåne County, Malmö, Sweden; 8 Department of Clinical Sciences, Lund University, Helsingborg, Sweden; 9 Department of Internal Medicine, Sahlgrenska Academy, University of Gothenburg, Gothenburg, Sweden

**Keywords:** Muscarinic cholinergic antagonists, Anticholinergic, Anticholinergic burden, Sweden, Anticholinergic adverse effects, Anticholinergic scales, Geriatrics, Cognitive impairment

## Abstract

**Background:**

Drugs with anticholinergic properties are associated with cognitive adverse effects, especially in patients vulnerable to central muscarinic antagonism. A variety of drugs show weak, moderate or strong anticholinergic effects. Therefore, the cumulative anticholinergic burden should be considered in patients with cognitive impairment. This study aimed to develop a Swedish Anticholinergic Burden Scale (Swe-ABS) to be used in health care and research.

**Methods:**

A systematic literature review was conducted in PubMed and Ovid Embase to identify previously published tools quantifying anticholinergic drug burden (i.e., exposure). Drugs and grading scores (0–3, no to high anticholinergic activity) were extracted from identified lists. Enteral and parenteral drugs authorized in Sweden were included. Drugs with conflicting scores in the existing lists were assessed by an expert group. Two drugs that were not previously assessed were also added to the evaluation process.

**Results:**

The systematic literature search identified the following nine anticholinergic burden scales: Anticholinergic Activity Scale, Anticholinergic Burden Classification, updated Anticholinergic Cognitive Burden scale, Anticholinergic Drug Scale, Anticholinergic Load Scale, Anticholinergic Risk Scale, updated Clinician-rated Anticholinergic Scale, German Anticholinergic Burden Scale and Korean Anticholinergic Burden Scale. A list of drugs with significant anticholinergic effects provided by The Swedish National Board of Health and Welfare was included in the process. The suggested Swe-ABS consists of 104 drugs scored as having weak, moderate or strong anticholinergic effects. Two hundred and fifty-six drugs were listed as having no anticholinergic effects based on evaluation in previous scales. In total, 62 drugs were assessed by the expert group.

**Conclusions:**

Swe-ABS is a simplified method to quantify the anticholinergic burden and is easy to use in clinical practice. Publication of this scale might make clinicians more aware of drugs with anticholinergic properties and patients’ total anticholinergic burden. Further research is needed to validate the Swe-ABS and evaluate anticholinergic exposure versus clinically significant outcomes.

**Supplementary Information:**

The online version contains supplementary material available at 10.1186/s12877-023-04225-1.

## Background

Acetylcholine is a neurotransmitter used by all cholinergic neurons in the central and peripheral nervous systems. It plays an important role in cognitive functions, such as memory processes [1]. Medications with anticholinergic properties (i.e. muscarinic cholinergic antagonists) are associated with a high risk of both central adverse effects, such as cognitive impairment, and peripheral adverse effects, such as dryness of the mouth and urinary retention [2]. These adverse effects may occur when anticholinergic drugs block acetylcholine binding to muscarinic receptors M1–M5, all of which have similar structures at their ligand-binding sites. The development of subtype-selective antagonists is one approach to diminish these adverse effects. Hence, the introduction of M3-selective urinary spasmolytics such as solifenacin and darifenacin has improved the treatment of urinary incontinence as they exert less central anticholinergic effects. Beyond urinary incontinence drugs with anticholinergic properties are used to treat a wide variety of medical conditions, including pain, sleep disorders, parkinsonism and depression [3].

Besides medications used particularly for their anticholinergic properties, there are medications with varying degrees of anticholinergic activity (AA), leading to non-intended anticholinergic effects. The cumulative effect of taking multiple medications with AA is known as anticholinergic burden and is associated with an increased risk of significant anticholinergic adverse drug reactions [4, 5].

Drugs with high anticholinergic potency are regarded as potentially inappropriate in treating older people, especially those with Alzheimer’s or other neurodegenerative diseases, because of conditions such as degeneration of cholinergic neurons in the basal forebrain or increased permeability of the blood–brain barrier [6]. However, recent studies suggested that even individuals aged ≤75 years [7], younger patients with Parkinson’s disease [8] and middle-aged people with Alzheimer’s disease [9] are at increased risk of anticholinergic adverse effects. Hence, individual vulnerability pertaining to neurodegenerative diseases, besides age, might play an important role in the risk of anticholinergic adverse effects [10].

The Swedish National Board of Health and Welfare has issued a list of drugs with significant anticholinergic effects that should be avoided in treating the elderly [11]. The total prescription of these listed drugs has decreased radically among the elderly in Sweden. Nevertheless, 3.8% of people aged ≥75 are prescribed these medications [12].

Currently, the following two major methods to assess a patient’s anticholinergic burden are available: (1) serum radioreceptor anticholinergic activity assay and (2) using expert-based lists of medications with anticholinergic properties [13]. The latter method has been suggested as the only clinically useful method [14]. Summer’s method using the drug risk number, which estimated the risk of drug-induced delirium, was the first clinical research method to be published [15]. Over the years, several expert-based lists have been developed, with most of them categorizing anticholinergic medications into groups based on their level of AA. However, they differ in the number and selection of included drugs as well as in the rating of AA [16].

In 2013, Duran et al. developed the first comprehensive list of drugs with anticholinergic properties based on seven published risk scales [17]. This method was later used to develop drug lists for countries such as Germany and South Korea [18, 19]. Adapting lists for specific countries is important because medication availability and prescribing patterns differ among countries [16].

Therefore, we aimed to develop a scale with drugs authorized and available in Sweden, using the experiences gained elsewhere. To our knowledge, a Swedish version of a scale for quantifying anticholinergic burden has not yet been introduced. However, a web-based risk assessment tool, Janusmed^®^, was available for two specific regions in Sweden at the start of the present study. Nevertheless, no Swedish expert-based list was available to us.

## Methods

### Search strategy and selection criteria

PubMed was searched for systematic reviews of previously published tools quantifying anticholinergic drug burden (i.e., exposure). To be classified as a systematic review, the study had to be planned and reported in accordance with the Preferred Reporting Items for Systematic Reviews and Meta-Analyses statement [20, 21]. The search was conducted in April 2020 with no date restriction. The search terms were as follows: anticholinergic [Title/Abstract] AND burden [Title/Abstract] AND scale [Title/Abstract] OR list [Title/Abstract] OR score [Title/Abstract] OR tool [Title/Abstract] AND review. The same procedure was used for Ovid Embase to ensure that all relevant reviews were found. In addition, the reference lists of the selected studies were searched manually for more studies. Furthermore, studies were included if they were (1) systematic reviews on tools to quantify anticholinergic drug burden or original research papers presenting anticholinergic risk scales and (2) written in English. Tools were included if they (1) provided medication lists with grading scores to quantify anticholinergic burden; (2) were based on clinical expert opinions and included drugs authorized in Sweden not previously assessed and (3) provided lists that were comparable to other lists. Tools based on equations were not included. The titles and abstracts of the identified studies were screened, and full-text articles were evaluated in case of uncertainty. To find any new lists with drugs not previously assessed, the search was updated in March 2022.

### Data extraction

The method established by Duran et al. and adapted by Kiesel et al. was used to construct a Swedish anticholinergic burden scale [17, 18]. Enteral and parenteral drugs were included. Drugs authorized and available in Sweden at the start of the present study (April 2020) were included. In addition, two clinically relevant substances, propiomazine and vortioxetine, were added.

Each drug was generally assigned a score from 0 (no AA) to 3 (high AA) in the identified lists why this gradation was chosen for this scale. However, the grading system differed in one list, the Anticholinergic Activity Scale, where a 5-point grading system (0–4) was used [22]. Therefore, this list was modified to 0–3 in accordance with Duran et al.’s methodology [17].

The agreement of the scores for each drug was evaluated. The algorithm employed by Kiesel et al. was used. If a drug was scored by ≥2 lists and there was agreement among the list scores, the drug was assigned that score. If a drug was scored by ≥2 lists with only a 1-point difference, the drug was assigned the higher score. If a drug was scored exclusively 0 in at least one existing list, the drug was scored 0. Further evaluation was needed (1) when the scores differed by ≥2 points between the lists and (2) if the drug was evaluated and scored 1–3 in only one list [18]. In addition, further evaluation was needed when (1) a drug was scored unanimously 3 by ≥2 lists but was not included on the list of medications with significant anticholinergic effects provided by the National Board of Health and Welfare [11] and (2) a drug was included on the abovementioned list but not included or scored 0–2 in the identified lists.

An expert group consisting of four physicians (two senior neuropsychiatrists, a senior general practitioner and a resident in both clinical pharmacology and psychiatry) and one clinical pharmacist was formed to further evaluate the selected drugs. To rate the mechanism of action of the drugs, central and peripheral anticholinergic adverse effects reported by Tune [4] were retrieved from the online version of DRUGDEX^®^. Any information about the muscarinic binding affinity of the drugs was retrieved from the Psychoactive Drug Screening Program K_i_ Database^®^ and DrugBank Online^®^, while Chew’s list was used for any information about a drug’s serum AA [23]. Additional sources of information, such as UpToDate^®^ and Martindale^®^, were used if information about a drug was absent, poor or outdated in DRUGDEX^®^. The Swedish summary of product characteristics for each drug was searched for contraindicated medical conditions, such as glaucoma, myasthenia gravis and benign prostatic hyperplasia. A score of 0 to 3 was assigned for each drug individually by the experts based on the mechanism of action, contraindications, frequency of adverse effects and type of adverse effects. The scoring was then discussed in the expert group leading to consensus and a final anticholinergic score. Information regarding the muscarinic binding affinity of the drugs was considered in the final discussion when available. In case of disagreement in the expert group, other physicians were consulted. The clinical experience of the expert group was considered in the rating process.

## Results

**Fig. 1:**
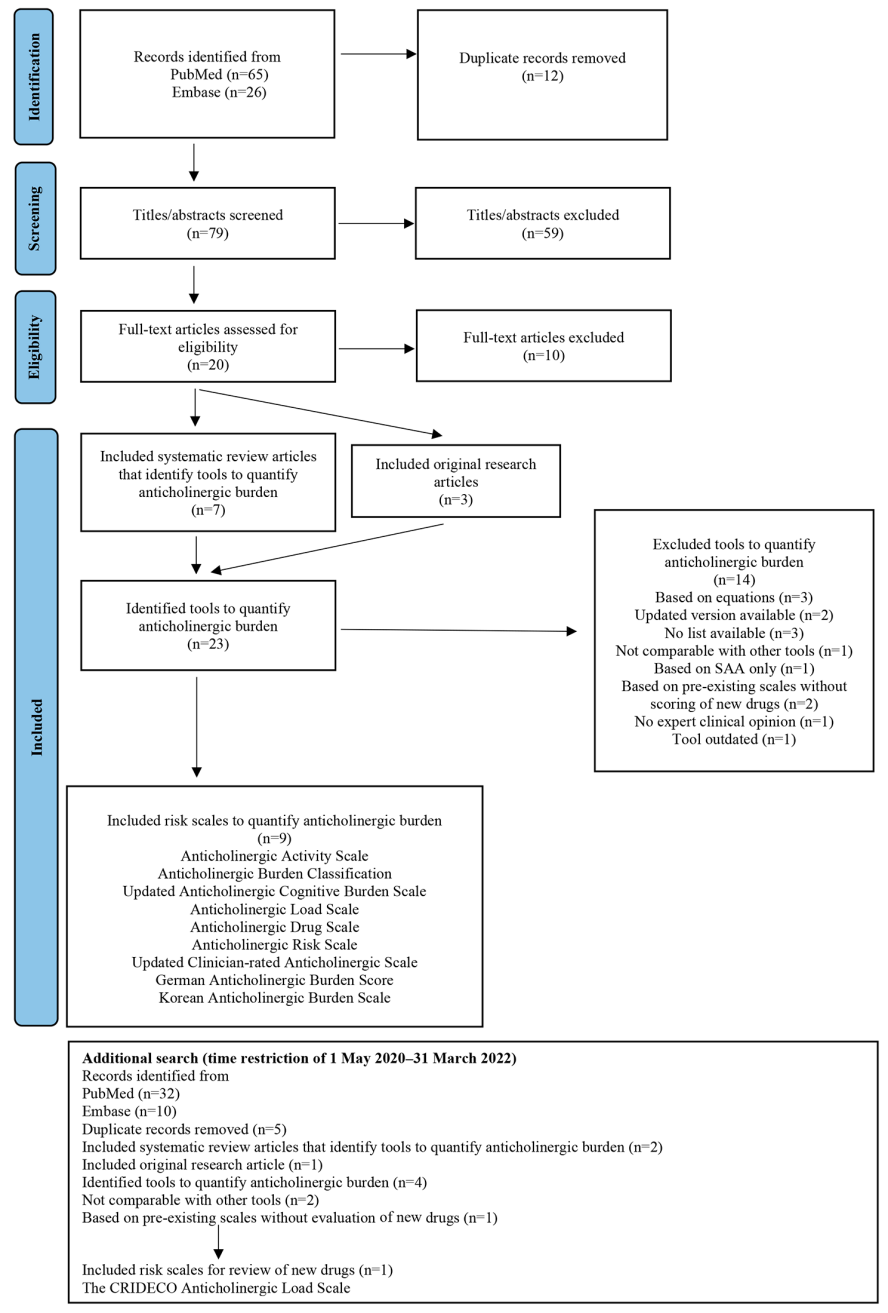
Preferred Reporting Items for Systematic Reviews and Meta-Analyses flow chart of the selection strategy

The search in PubMed and Ovid Embase resulted in seven systematic reviews [17, 20, 24–28] and two original research articles [19, 29]. The following nine anticholinergic risk scales met the inclusion criteria: Anticholinergic Activity Scale, Anticholinergic Burden Classification, updated Anticholinergic Cognitive Burden scale, Anticholinergic Drug Scale, Anticholinergic Load Scale, Anticholinergic Risk Scale, updated Clinician-rated Anticholinergic Scale, German Anticholinergic Burden Scale and Korean Anticholinergic Burden Scale [18, 19, 22, 30–35]. A flowchart of the selection strategy is presented in Fig. 1 [21]. The list of medications with significant anticholinergic effects provided by the National Board of Health and Welfare was then added to these scales [11]. Excluded tools with the reasons for exclusion are presented in Table 1.

An additional database search with a time restriction of 1 May 2020 to 31 March 2022 yielded two new reviews [13, 36], one new original research article [37] and four additional scales (the Delirogenic Risk Scale [38], modified Anticholinergic Burden Scale [39], Anticholinergic Toxicity Score [40] and CRIDECO Anticholinergic Load Scale [37]; Fig. 1). The Delirogenic Risk Scale and the Anticholinergic Toxicity Score were not comparable with other scales [38, 40]. The modified Anticholinergic Burden Scale was produced by combining pre-existing scales without any evaluation of new drugs [39]. The CRIDECO Anticholinergic Load Scale was based on risk scales already included in the assessment. However, it included one enteral drug authorized in Sweden previously not evaluated in the existing scales [37]. This drug was assessed by the expert group.

A total of 234 drugs scored 1–3 were extracted from the existing lists and the list of drugs with definite anticholinergic effects provided by the National Board of Health and Welfare. Of these, 107 drugs were excluded because they were not authorized in Sweden (Additional file 1 Table 1). Furthermore, nine drugs were excluded due to other modes of administration than enteral or parenteral (Additional file 1 Table 2). Six drugs were then added: two of them were previously not rated (propiomazine and vortioxetine) and four of them scored 0 in previous scales (chlorzoxazone, melperone, memantine and sertindole). A summary of the remaining 124 drugs is presented in Table 2.

**Table 1:** Excluded tools with the reason for exclusion

Tool	Reason for exclusion
Muscarinic Acetylcholinergic Receptor ANTagonist Exposure Scale [41]	Based on equations
Drug Burden Index [42]	
Drug Burden Index - Anticholinergic component [43]	
Aizenberg’s Anticholinergic Burden Scale [44]	No list available
Cancelli’s Anticholinergic Burden Scale [45]	
Whalley’s Anticholinergic Burden Scale [46]	
Anticholinergic Cognitive Burden Scale [5]	Updated versions published
Clinician-rated Anticholinergic Score [47]	
Chew’s list [23]	Classification based only on SAA
Salahudeen’s Anticholinergic Burden Scale [24]	The scale was produced by combining pre-existing scales without any scoring of new drugs
Brazilian Anticholinergic Activity Drug Scale [29]	The scale was produced by combining pre-existing scales without any scoring of new drugs authorized and available in Sweden
Anticholinergic Effect on Cognition [48]	Expert clinical opinion was missing
Summer’s Drug Risk Number [15]	The tool was outdated
Clinical Index, Pharmacological Index [49]	The tool was not comparable with other tools

**Table 2:** Summary of drugs scored 1-3 and extracted from the existing scales and drugs added by the expert group

Drug	ATC code	Han [32]	Ancelin [31]	Carnahan [30]	Boustani [34]	Rudolph [35]	Ehrt [22]	Sittironnarit [33]	Kiesel [18]	Jun [19]	Duran [17]
Alimemazine*	R06AD01		2		1		0			1	Low
Alprazolam	N05BA12	1	3	1	1			1	1	1	Disc (high)
Amantadine	N04BB01			1	2	2			2	2	Low
Amitriptyline*	N06AA09	3	3	3	3	3	3	3	3	3	High
Ampicillin	J01CA01			1					1	0	Disc (low)
Aripiprazole	N05AX12				1			0	1	1	
Asenapine	N05AH05				1				1		
Atenolol	C07AB03	1		0	1		0	0	1	0	0
Atropine*	A03BA01	3		3	3	3		3	3	3	High
Azathioprine	L04AX01			1					1	0	Disc (low)
Baclofen	M03BX01	2		0		2			1	1	Low
Biperiden*	N04AA02									3	
Bisacodyl	A06AB02			0				1	1	0	Disc (low)
Bromocriptine	G02CB01			1			0		1	0	Low
Bupropion	N06AX12	1		0	1				1	1	Disc (low)
Captopril	C09AA01			1	1		0	0	1	0	Disc (low)
Carbamazepine	N03AF01	1		2	2		0	0	2	1	Low
Celecoxib	M01AH01			0				1	1	0	Disc (low)
Cetirizine	R06AE07	2		0	1	2		2	1	1	Low
Chlorprothixene*	N05AF03						0			3	
Chlorthalidone	C03BA04			1	1			0	1	0	Disc (low)
Chlorzoxazone	M03BB03			0						0	
Ciclosporin	L04AD01			1					1	0	Disc (low)
Cinnarizine	N07CA02									1	
Citalopram	N06AB04			0			1	1	1	1	Low
Clemastine*	R06AA04			3	3				3	3	High
Clindamycin	J01FF01			1					1	0	Disc (low)
Clomipramine*	N06AA04		3	3	3				3	3	High
Clonazepam	N03AE01			1				1	1	1	Low
Clozapine*	N05AH02			3	3	2	3		3	3	High
Codeine	R05DA04	1	2	1	1		0	1	1	1	Low
Colchicine	M04AC01		3	0	1			0		0	Disc (high)
Darifenacin*	G04BD10			3	3				3		High
Desloratadine	R06AX27				1				1	1	
Dexamethasone	H02AB02			1				0	1	0	Disc (low)
Diazepam	N05BA01	1		1	1		1	1	1	1	Low
Digoxin	C01AA05		3	1	1		1	1	1	1	Disc (high)
Diltiazem	C08DB01			1				0	1	0	Disc (low)
Dimenhydrinate*	R06AA02			3	3				3	3	High
Diphenhydramine	R05CA10	3		3	3	3			3	3	High
Dipyridamole	B01AC07			1	1		0		1	0	Disc (low)
Domperidone	A03FA03							1	1	0	Low
Entacapone	N04BX02			0		1			1	0	Low
Escitalopram	N06AB10			0				1	1	1	Disc (low)
Etoricoxib	M01AH05								1		
Famotidine	A02BA03			1				0	1	0	Disc (low)
Fentanyl	N02AB03			1	1			0	1	1	Low
Fesoterodine*	G04BD11				3				3	3	
Fexofenadine	R06AX26	2		0	0			2	1	0	Low
Fluoxetine	N06AB03	1		1			1	1	1	1	Low
Flupentixol	N05AF01									1	
Fluvoxamine	N06AB08			1	1		1	1	1	1	Low
Furosemide	C03CA01		3	1	1		1		1	1	Disc (high)
Gentamicin	J01GB03			1					1	0	Disc (low)
Glycopyrronium^inj^*	A03AB02									2	
Guaifenesin	R05CA03	1		0					1	1	Discr (low)
Haloperidol	N05AD01			0	1	1	0	2	2	1	Low
Hydralazine	C02DB02			1	1			0	1	1	Disc (low)
Hydrocortisone	H02AB09			1	1			0	1	1	Disc (low)
Hydroxyzine*	N05BB01		3	3	3	3			3	3	High
Hyoscyamine*	A03BA03			3	3	3				3	High
Isosorbide mononitrate	C01DA14			1				0	1		Disc (low)
Ketorolac	M01AB15	1							1	0	Low
Lansoprazole	A02BC03			0			1	0	1	0	Disc (low)
Levodopa	N04BA01	1		0		1		1	1		0
Levomepromazine*	N05AA02		3	2	2				3	2	High
Lithium	N05AN01			0				1	1	0	Low
Loperamide	A07DA03	1		1	1	2		1	2	1	Low
Loratadine	R06AX13	1		0	1	2		1	1	1	Low
Lorazepam	N05BA06			1					1	1	Disc (low)
Meclizine*	R06AE05			3	3	3				3	High
Melperone	N05AD03						0		0		
Memantine	N06DX01							0	0	0	
Metformin	A10BA02			0				1	1	0	Disc (low)
Methadone	N07BC02	2							2		Low
Methotrexate	L04AX03			0				1	1	0	Disc (low)
Methylprednisolone	H02AB04			1				0	1	0	Disc (low)
Metoclopramide	A03FA01	3		0		1	0	1	1	0	Disc (high)
Metoprolol	C07AB02	1		0	1		0	0	1	0	0
Midazolam	N05CD08			1					1	1	Disc (low)
Mirtazapine	N06AX11			0		1			1	1	Low
Morphine	N02AA01	1		1	1				1	1	Low
Naratriptan	N02CC02							1	1	0	Disc (low)
Nifedipine	C08CA05			1	1		0	0	1	0	0
Nortriptyline*	N06AA10	3		3	3	2	2		3	3	High
Olanzapine	N05AH03	1		1	3	2	2		2	3	Low
Orphenadrine*	M03BC01		3	3	3		3		3	3	High
Oxazepam	N05BA04			1			0	1	1		Disc (low)
Oxcarbazepine	N03AF02			2	2				2	2	Low
Oxybutynin*	G04BD04		3	3	3	3	3	2	3	3	High
Oxycodone	N02AA05	1		1				1	1	1	Low
Paliperidone	N05AX13				1				1	1	
Paroxetine	N06AB05	2		1	3	1	2	2	2	2	Low
Perphenazine	N05AB03	2		1	3	3	0		1	2	Disc (high)
Pethidine	N02AB02			2	2		0		2	2	Low
Phenobarbital	N03AA02	1		0			1		1	0	Disc (low)
Piperacillin	J01CA12			1					1	0	Disc (low)
Pramipexole	N04BC05			0		1	0		1	0	Disc (low)
Prednisolone	H02AB06			1			0	0	1	1	Disc (low)
Prochlorperazine*	N05AB04	2		1		2	0	2			Low
Promethazine*	R06AD02			3	3	3		0	1		High
Propiomazine	N05CM06										
Quetiapine	N05AH04	2		0	3	1	1		2	2	Low
Ranitidine	A02BA02	2		2	1	1	1	1	2	1	Low
Risperidone	N05AX08	1		0	1	1		1	1	1	Low
Rotigotine^patch^	N04BC09								1		
Scopolamine*	A04AD01	3		3	3				3	3	High
Selegiline	N04BD01			0		1	0		1	0	Disc (low)
Sertindole	N05AE03									0	
Sertraline	N06AB06	1		1			0	0	1	0	0
Solifenacin*	G04BD08				3			0	3	3	
Sumatriptan	N02CC01							1	1	0	Disc (low)
Theophylline	R03DA04		2	1	1		1	2	2	1	Low
Tolterodine*	G04BD07	3		3	3	2		3	3	3	High
Tramadol	N02AX02	2		1				2	2	2	Low
Triamcinolone	H02AB08			1				0	1	0	Disc (low)
Valproic acid	N03AG01			1				0	1	0	Disc (low)
Vancomycin	J01XA01			1					1	0	Disc (low)
Venlafaxine	N06AX16	1		0	1		0	1	1	1	0
Vortioxetine	N06AX26										
Warfarin	B01AA03			1	1		0	0	1	0	0
Ziprasidone	N05AE04					1			1	1	Disc (low)
Zolmitriptan	N02CC03							1	1	0	Disc (low)
Zuclopenthixol	N05AF05									2	

* On the list of drugs with definite anticholinergic effects provided by the Swedish National Board of Health and Welfare [11]

High, high potency anticholinergics; Low, low potency anticholinergics; Disc (high), high discrepancy in drugs with high scores; Disc (low), low discrepancy in drugs with low grades

In total, 62 drugs, including two drugs previously not assessed and one drug from the second database search, were evaluated by the expert group. The rating was reconsidered for some drugs within the same drug class, based on the Anatomical Therapeutic Chemical Classification by the World Health Organization, that were scored differently [18]. The rating could also be reconsidered for structurally alike drugs that were assigned different scores.

Twenty-one drugs were assessed by the expert group as having no anticholinergic effects (score 0; Table 3), while 256 drugs were listed as having no anticholinergic effects based on their assessment in previous scales [18] (Additional file 1 Table 3).

**Table 3:** Drugs assessed by expert group as having no anticholinergic effects

Score 0
Ampicillin
Azathioprine
Celecoxib
Chlorzoxazone
Ciclopsporin
Clindamycin
Colcichine
Domperidone
Etoricoxib
Fexofenadine
Furosemide
Gentamicin
Ketorolac
Melperone
Memantine
Metoclopramide
Naratriptan
Piperacillin Rotigotine^patch^
Sumatriptan
Vancomycin

The suggested final Swedish Anticholinergic Burden Scale (Swe-ABS) is presented in Table 4. It presents 23 drugs with strong anticholinergic effects (score 3), 16 drugs with moderate anticholinergic effects (score 2) and 65 drugs with low anticholinergic effects (score 1).

**Table 4:** The Swedish Anticholinergic Burden Scale (Swe-ABS)

Score 1		Score 2	Score 3
Alprazolam*	Levodopa	Alimemazine*	Amitriptyline
Aripiprazole	Lithium*	Amantadine*	Atropine
Asenapine*	Loratadine*	Carbamazepine*	Biperiden*
Atenolol	Lorazepam*	Flupentixol*	Chlorprothixene*
Baclofen *	Metformin	Loperamide	Clemastine
Bisacodyl	Methadone*	Olanzapine*	Clomipramine
Bromocriptine	Methotrexate	Oxcarbazepine	Clozapine
Bupropion	Methylprednisolone	Paroxetine*	Darifenacin
Captopril	Metoprolol	Perphenazine*	Dimenhydrinate
Cetirizine*	Midazolam*	Pethidine*	Diphenhydramine*
Chlorthalidone	Mirtazapine	Propiomazine*	Fesoterodine
Cinnarizine*	Morphine*	Quetiapine*	Glycopyrronium^inj^*
Citalopram*	Nifedipine	Ranitidine	Hydroxyzine
Clonazepam	Oxazepam	Theophylline	Hyoscyamine
Codeine*	Oxycodone	Tramadol	Levomepromazine
Desloratadine*	Paliperidone *	Zuclopenthixol*	Meclizine
Dexamethasone	Phenobarbital		Nortriptyline
Diazepam	Pramipexole		Orphenadrine
Digoxin*	Prednisolone		Oxybutynin
Diltiazem	Prochlorperazine*		Promethazine*
Dipyridamole	Risperidone		Scopolamine
Entacapone	Selegiline		Solifenacin*
Escitalopram*	Sertindole *		Tolterodine
Famotidine	Sertraline		
Fentanyl	Tapentadol*		
Fluoxetine	Triamcinolone		
Fluvoxamine	Valproic acid		
Guaifenesin	Venlafaxine		
Haloperidol *	Vortioxetine*		
Hydralazine	Warfarin		
Hydrocortisone	Ziprasidone*		
Isosorbide mononitrate Lansoprazole	Zolmitriptan*		

*Evaluated by the expert group

## Discussion

Several anticholinergic burden scales have been published. However, no international standard scale has been recommended for the quantification of anticholinergic burden [36]. The Swe-ABS was developed from nine existing anticholinergic risk scales [18, 19, 22, 30–35] and the list of medications with significant anticholinergic effects provided by the National Board of Health and Welfare [11]. Although grey literature was not searched for possible unpublished anticholinergic risk scales, several published systematic reviews have been reviewed in this study to reduce the risk of missing existing scales. Most of the included scales are over a decade old and lack drugs marketed after the publication of the scales [22, 30–35]. However, two of the scales were newly published and updated with new drugs [18, 19], which makes the Swe-ABS up to date. Furthermore, drugs not authorized in Sweden were excluded and two new drugs were added to adapt the scale to the Swedish market.

In the scoring process, the algorithm employed by Kiesel et al. to develop the German Anticholinergic Burden Score was used [18]. In a study assessing the quality of published anticholinergic burden scales, the German scale achieved the highest percentage in quality, together with the updated Anticholinergic Cognitive Burden scale [13]. Furthermore, a newly published study showed that the German Anticholinergic Burden Score appears to be comparable with the validated Anticholinergic Drug Scale regarding the effect of an anticholinergic burden on cognitive function [50]. Contrary to Kiesel et al., the authors of this study also considered muscarinic binding affinity in the assessment of drugs that needed further evaluation.

The present study has some limitations. First, the dependability of the reported adverse effects as a measure is contingent on both frequency and reliability. For example, an absence of reported anticholinergic adverse effects may be because they are absent or underreported. Other issues include how anticholinergic and antihistaminic effects should be distinguished and the challenging differentiation between anticholinergic side effects and the mechanism of action of an assessed drug. Moreover, information about the muscarinic binding affinity was lacking for several drugs and for others, the reported dissociation constant differed significantly between studies. The expert group consisted of people with extensive clinical experience from pharmaceutical-intensive specialties contributing to a multifaceted assessment. However, making a completely impartial assessment regarding anticholinergic adverse effects based on expert clinical opinion may not be possible [48].

Second, in cases where a drug had been assessed using ≥2 scales and the scores differed by only 1 point, the drug was automatically assigned the higher score based on Kiesel et al.’s methodology [18]. This might have resulted in an overestimation of the AA of the drug [19]. However, the risk of missing any drug with anticholinergic properties was reduced [18]. Furthermore, if a drug was scored by ≥2 lists and there was agreement among the list scores, the drug was assigned that score. This might have resulted in repetition of incorrectly assessed drugs. The gradation 0–3 was selected to make this scale comparable with several existing scales. A wider scoring range might have been valuable in distinguishing the drugs even more [24]. On the other hand, this could create an illusion of that it is a more precise measure of anticholinergic effects than it is.

Third, as in the case of many previously published scales, this scale does not consider dosage even though anticholinergic effects are considered dose-dependent [6, 23, 41, 51]. Whether an equal anticholinergic score results in the same effect has been questioned. For example, whether three drugs with an anticholinergic score of 1 are equivalent to one drug with an anticholinergic score of 3 [26]. This limits the application in clinical practice. Moreover, there is no advice on how to apply the scale in relation to changes in medication at a specific cut off value for high anticholinergic burden [6]. It is also recommended that individual vulnerability is to be considered, which adds a further level of complexity and uncertainty. Furthermore, variables such as drug–drug interactions and possible development of tolerance for anticholinergic drug effects are yet to be considered [6].

Fourth, this list is not comprehensive. Drugs with routes of administration other than enteral or parenteral were not included in this list due to incomplete data on systemic effects, and the list does not include all drugs marketed in Sweden. This scale is proposed to be used as a guideline when evaluating anticholinergic burden in patients, especially in those vulnerable to central muscarinic antagonism. Nevertheless, both central and peripheral anticholinergic adverse effects have been assessed in this study due to increased permeability of the blood–brain barrier in the elderly and individuals with neurodegenerative diseases [6, 52].

The authors encourage a possible adaptation of Swe-ABS for the other Nordic countries with comparable treatment guidelines and medication availability. To avoid repetition of previous assessment, the Swe-ABS could be modified for medications approved in other Nordic countries. This approach would be timesaving, although also involve a significant risk that the list does not cover approved drugs in the country for which it is intended. In the adaptation process we therefore also recommend review of excluded drugs in this paper (Additional file 1 Table 1). We encourage a Nordic cross-national collaboration regarding validation studies in different clinical settings and assessment of new drugs on the market. The use of an adapted Swe-ABS in other Nordic countries with similar healthcare systems could contribute to increased awareness of drugs with anticholinergic properties and reduce the overall anticholinergic burden. However, mentioned limitations, specifically the lack of dose-related information must be considered.

During the finalization of this article, the web-based risk assessment tool Janusmed^®^, which was earlier available in two county councils in Sweden, became available throughout Sweden. In the future, it would be useful to review the coherence between these two tools. Swe-ABS will be validated in an ongoing study conducted at a memory clinic in southern Sweden. We aim to establish a continuously updated tool for quantifying anticholinergic burden that is clinically relevant. An eHealth application integrated with electronic medical record systems could be one way to automize the use of this scale.

## Conclusions

The Swe-ABS is a simplified method to quantify anticholinergic burden and it is easy to use in clinical practice. Publication of this scale might make clinicians more aware of drugs with anticholinergic properties and patients’ total anticholinergic burden. Further research is needed to validate the Swe-ABS and evaluate anticholinergic exposure versus clinically significant outcomes.

### Electronic supplementary material

Below is the link to the electronic supplementary material.

Supplementary Material 1

## Data Availability

All data generated or analysed during this study are included in this published article (and its additional files).

## Abbreviations

AA: Anticholinergic activity
Swe-ABS: Swedish Anticholinergic Burden Scale
