# Supplementary material for: Development of the Swedish anticholinergic burden scale (Swe-ABS)
Source: BMC Geriatr. 2023 Aug 25;23:518. doi: 10.1186/s12877-023-04225-1 (PMC10464171; doi:10.1186/s12877-023-04225-1)
Supplement: Supplementary file 1 — Supplementary Material 1 [file 12877_2023_4225_MOESM1_ESM.docx]

# ST1 Drugs excluded from the previous tools because they were not authorized in Sweden (scored 1–3 in existing scales)

| Acepromazine |
| --- |
| Alverine |
| Amisulpride |
| Amoxapine |
| Belladonna |
| Benazepril |
| Benzatropine |
| Blonanserin |
| Bromperidol |
| Brompheniramine |
| Buclizine |
| Carbinoxamine |
| Carisoprodol |
| Cefamandole |
| Cefoxitin |
| Cephalothin |
| Chlordiazepoxide |
| Chlorpheniramine (chlorphenamine) |
| Chlorpromazine |
| Cimetidine |
| Cimetropium |
| Clidinium |
| Cloperastine |
| Clorazepate |
| Cortisone |
| Cyclobenzaprine |
| Cycloserine |
| Cyproheptadine |
| Desipramine |
| Desvenlafaxine |
| Dexbrompheniramine |
| Dexchlorpheniramine |
| Dextromethorphan |
| Dicyclomine (dicycloverine) |
| Difemerine |
| Difenidol |
| Digitoxin |
| Dimetindene |
| Disopyramide |
| Divalproex |
| Dothiepin |
| Doxepin |
| Doxylamine |
| Emepronium |
| Estazolam |
| Flavoxate |
| Flunitrazepam |
| Fluphenazine |
| Flurazepam |
| Homochlorcyclizine |
| Homatropine |
| Hydrocodone |
| Iloperidone |
| Imidafenacin |
| Imipramine |
| Isosorbide |
| Isosorbide dinitrate |
| Levocetirizine |
| Lumiracoxib |
| Maprotiline |
| Mebeverine |
| Mequitazine |
| Methocarbamol |
| Molindone |
| Nefazodone |
| Nefopam |
| Nizatidine |
| Octylonium (otilonium) bromide |
| Opipramol |
| Oxapium iodide (cyclonium) |
| Pancuronium |
| Phenelzine |
| Pheniramine |
| Pimozide |
| Piprinhydrinate (diphenylpyraline) |
| Prednisone |
| Pridinol |
| Procyclidine |
| Promazine |
| Propantheline |
| Propiverine |
| Propoxyphene (dextropropoxyphene) |
| Protriptyline |
| Pseudoephedrine |
| Pyrilamine (mepiramine) |
| Quinidine |
| Temazepam |
| Thiethylperazine |
| Thioridazine |
| Thiothixene |
| Tiemonium |
| Timepidium |
| Tiquizium |
| Tizanidine |
| Trandolapril |
| Trazodone |
| Triamterene |
| Triazolam |
| Triﬂuoperazine |
| Trihexyphenidyl |
| Trimebutine |
| Trimipramine |
| Triprolidine |
| Tropatepine |
| Trospium |
| Valethamate bromide |
| Zotepine |

**ST2** Drugs not included due to modes of administration other than enteral or parenteral

| Aclidinium (inhalative) |
| --- |
| Betaxolol (ophthalmic) |
| Emedastine (ophthalmic) |
| Fluticasone-salmeterol (inhalative) |
| Glycopyrronium (inhalative) |
| Ipratropium (inhalative) |
| Ketotifen (ophthalmic) |
| Loxapine (inhalative) |
| Tiotropium (inhalative) |

**ST3** Drugs listed as having no anticholinergic effects based on assessment in previous scales (drugs scored 0 in existing scales, enteral and parenteral modes of administration and authorized in Sweden)

| Acarbose |
| --- |
| Acetazolamide |
| Acetylcysteine |
| Acetylsalicylic acid |
| Acitretin |
| Activated charcoal |
| Acyclovir |
| Adenosine |
| Adrenaline |
| Agomelatine |
| Alendronate |
| Allopurinol |
| Aluminium salts |
| Amiloride |
| Amiodarone |
| Amlodipine |
| Amoxicillin |
| Amoxicillin-Clavulanate |
| Anagrelide |
| Anastrozole |
| Apixaban |
| Ascorbic acid |
| Atorvastatin |
| Azithromycin |
| Betahistine |
| Betaine |
| Bicalutamide |
| Bisoprolol |
| Bromhexine |
| Budesonide |
| Bumetanide |
| Buprenorphine |
| Buspirone |
| Cabergoline |
| Caffeine |
| Calcitonin |
| Calcitriol |
| Calcium carbonate |
| Candesartan |
| Carvedilol |
| Cefazolin |
| Ceftriaxone |
| Cefuroxime |
| Chlorambucil |
| Cholestyramine |
| Cilostazol |
| Ciprofloxacin |
| Clarithromycin |
| Clavulanate |
| Clobazam |
| Clodronate |
| Clomethiazole |
| Clonidine |
| Clopidogrel |
| Clotrimazole |
| Cloxacillin |
| Cholecalciferol |
| Colestipol |
| Conjugated estrogens |
| Cyanocobalamin |
| Cyclophosphamide |
| Dabigatran |
| Darbepoetin |
| Deferasirox |
| Desmopressin |
| Dextran |
| Diclofenac |
| Disulfiram |
| Dobutamine |
| Donepezil |
| Doxazosin |
| Doxycycline |
| Dulaglutide |
| Duloxetine |
| Dydrogesterone |
| Ebastine |
| Edoxaban |
| Empagliflozin |
| Enalapril |
| Enoxaparin |
| Eprosartan |
| Ergocalciferol |
| Erythromycin |
| Epoetin alfa |
| Esomeprazole |
| Estradiol |
| Estriol |
| Ethambutol |
| Ethinyl estradiol |
| Ezetimibe |
| Famciclovir |
| Felbamate |
| Felodipine |
| Fenofibrate |
| Ferrous sulfate |
| Filgrastim |
| Finasteride |
| Fish oil |
| Flecainide |
| Flucloxacillin |
| Fluconazole |
| Fludrocortisone |
| Flumazenil |
| Fluorouracil |
| Flutamide |
| Folic acid |
| Fosinopril |
| Gabapentin |
| Galantamine |
| Gemfibrozil |
| Glibenclamide |
| Glimepiride |
| Glucagon |
| Glucosamine |
| Goserelin acetate |
| Gramicidin |
| Guanfacine |
| Heparin |
| Histidine |
| Hydrochlorothiazide |
| Hydromorphone |
| Hydroxycarbamide |
| Hydroxychloroquine |
| Ibuprofen |
| Imipenem-Cilastatin |
| Insulin |
| Irbesartan |
| Isoniazid |
| Ketoprofen |
| Labetalol |
| Lactulose |
| Lamotrigine |
| Lercanidipine |
| Leuprolide |
| Levetiracetam |
| Levofloxacin |
| Levothyroxine |
| Lidocaine |
| Liothyronine |
| Lisinopril |
| Losartan |
| Lysine |
| Macrogol |
| Magnesium preparations |
| Mannitol |
| Medroxyprogesterone |
| Megestrol |
| Melatonin |
| Meloxicam |
| Mesalazine |
| Methenamine |
| Methylthioninium |
| Methylphenidate |
| Metolazone |
| Metronidazole |
| Mexiletine |
| Mianserin |
| Midodrine |
| Misoprostol |
| Moclobemide |
| Modafinil |
| Montelukast |
| Moxifloxacin |
| Moxonidine |
| Nabumetone |
| Naloxone |
| Naproxen |
| Nitrofurantoin |
| Nitroglycerin |
| Norepinephrine |
| Norethisterone |
| Nystatin |
| Octreotide |
| Olmesartan medoxomil |
| Omega-3 triglycerides |
| Omeprazole |
| Pamidronate |
| Pantoprazole |
| Paracetamol |
| Perindopril |
| Phenylephrine |
| Phenylpropanolamine |
| Phenytoin |
| Phytonadione |
| Pilocarpine |
| Pioglitazone |
| Piroxicam |
| Potassium chloride |
| Potassium citrate |
| Pravastatin |
| Probenecid |
| Progesterone |
| Propafenone |
| Propranolol |
| Protamine |
| Pseudoephedrine |
| Psyllium |
| Pyrazinamide |
| Pyridostigmine |
| Pyridoxine |
| Quinapril |
| Quinine |
| Rabeprazole |
| Raloxifene |
| Ramipril |
| Reboxetine |
| Repaglinide |
| Rifampicin |
| Risedronate |
| Rivaroxaban |
| Rivastigmine |
| Ropinirole |
| Rosuvastatin |
| Roxithromycin |
| Saccharomyces boulardii |
| Senna |
| Sevelamer |
| Sildenafil |
| Simethicone |
| Simvastatin |
| Sitagliptin |
| Sodium bicarbonate (hydrogen carbonate) |
| Sodium chloride |
| Sotalol |
| Spironolactone |
| Sterculia |
| Sucralfate |
| Sulfasalazine |
| Sulfamethoxazole |
| Succinylcholine (suxamethonium) |
| Tadalafil |
| Tamoxifen |
| Tamsulosin |
| Telmisartan |
| Terazosin |
| Terbinafine |
| Teriparatide |
| Testosterone |
| Tetracycline |
| Thiamazole |
| Thiamine |
| Tibolone |
| Tocopherol |
| Tolcapone |
| Topiramate |
| Torsemide |
| Trimethoprim |
| Ursodiol |
| Valsartan |
| Vardenafil |
| Varenicline tartrate |
| Vemurafenib |
| Verapamil |
| Zinc preparations |
| Zolpidem |
| Zopiclone |

# References

1. Aging Brain Care. Aging Brain Program: Anticholinergic Cognitive Burden Scale 2012 Update. 2012. https://gwep.med.ucla.edu/files/view/docs/initiative2/conferences/Anticholinergic-Burden-Scale.pdf. Accessed 31 Mar 2022.

2. Ancelin ML, Artero S, Portet F, Dupuy AM, Touchon J, Ritchie K. Non-degenerative mild cognitive impairment in elderly people and use of anticholinergic drugs: longitudinal cohort study. BMJ. 2006;332(7539):455-9.

3. Carnahan RM, Lund BC, Perry PJ, Pollock BG, Culp KR. The Anticholinergic Drug Scale as a measure of drug-related anticholinergic burden: associations with serum anticholinergic activity. J Clin Pharmacol. 2006;46(12):1481-6.

4. Ehrt U, Broich K, Larsen JP, Ballard C, Aarsland D. Use of drugs with anticholinergic effect and impact on cognition in Parkinson’s disease: a cohort study. J Neurol Neurosurg Psychiatry. 2010;81(2):160-5.

5. Han L, Agostini JV, Allore HG. Cumulative anticholinergic exposure is associated with poor memory and executive function in older men. J Am Geriatr Soc. 2008;56(12):2203-10.

6. Jun K, Hwang S, Ah YM, Suh Y, Lee JY. Development of an Anticholinergic Burden Scale specific for Korean older adults. Geriatr Gerontol Int. 2019;19(7):628-34.

7. Kiesel EK, Hopf YM, Drey M. An anticholinergic burden score for German prescribers: score development. BMC Geriatr. 2018;18(1):239.

8. Rudolph JL, Salow MJ, Angelini MC, McGlinchey RE. The Anticholinergic Risk Scale and anticholinergic adverse effects in older persons. Arch Intern Med. 2008;168(5):508-13.

9. Sittironnarit G, Ames D, Bush AI, Faux N, Flicker L, Foster J, et al. Effects of anticholinergic drugs on cognitive function in older Australians: results from the AIBL study. Dement Geriatr Cogn Disord. 2011;31(3):173-8.
